# Utilizing Data Quality Indices for Strategic Sensor Channel Selection to Enhance Performance of Hand Gesture Recognition Systems

**DOI:** 10.3390/s26041213

**Published:** 2026-02-12

**Authors:** Shen Zhang, Hao Zhou, Rayane Tchantchane, Gursel Alici

**Affiliations:** Applied Mechatronics and Biomedical Engineering Research (AMBER) Group, School of Engineering, University of Wollongong, Wollongong, NSW 2522, Australia; sz983@uowmail.edu.au (S.Z.); hzhou@uow.edu.au (H.Z.); rt910@uowmail.edu.au (R.T.)

**Keywords:** data quality index, channel selection, hand gesture recognition, multi-modal sensing, pressure-based force myography (pFMG), surface electromyography (sEMG)

## Abstract

This study proposes a data quality-driven channel selection methodology to improve hand gesture recognition performance in multi-channel wearable Human–Machine Interface (HMI) systems. The methodology centers around calculating (i) five data quality indices for both surface electromyography (sEMG) and pressure-based force myography (pFMG) signals and (ii) establishing a relationship between these data quality indices and the accuracy of gesture recognition for applications typified by prosthetic hand control. Machine learning (ML)-based and correlation-based methods were used to select three optimal channel/pair configurations from an eight-channel/pair system. Evaluations on the UOW and Ninapro DB2 datasets showed that the proposed methods consistently outperformed random channel selection, with the ML-based approach achieving the best results (76.36% for sEMG, 71.59% for pFMG, and 88.2% for fused sEMG-pFMG on the UOW dataset and 70.28% on Ninapro DB2). Notably, using three pairs of strategically selected sEMG-pFMG channels generated 88.2%, which is comparable to the 88.38% accuracy obtained with a full eight-channel sEMG system on the UOW dataset, highlighting the efficacy of our channel selection methodologies. These results highlight the value of data quality indices for sensor selection and provide a foundation for developing more efficient wearable HMI systems.

## 1. Introduction

Non-invasive and wearable Human–Machine Interface (HMI) systems are employed to decode users’ intentions or gestures for many applications where human and a machine are in the same loop to work/exist together. One important application is the upper-limb rehabilitation for which non-invasive systems are easier to implement with reduced safety concerns and fewer ethical issues [1,2], compared to invasive systems. These benefits have driven extensive research into diverse sensing modalities to obtain a realistic gesture recognition performance. By recording bio signals (muscle activities) of upper limbs, it is possible to identify the unique patterns underlying each specific movement/gesture, which can be employed to control prosthetic hands.

Due to limited space around the human arm, inadequate data, and restricted functionality in uni-modal sensing systems, co-located multi-modal sensing systems offer a potential solution [3,4]. Integrating complementary information from different sensing modalities offers a promising approach to overcome these challenges [1,5,6]. Additionally, co-located multi-modal sensing systems not only enhance the accuracy of hand gesture recognition but also maintain a similar wearable size compared to uni-modal sensing systems. Surface electromyography (sEMG) is the primary sensing system for the hand gesture recognition to control prosthetic hands [6,7]. This is due to its ability to directly capture motor unit action potential (MUAP) signals during muscle contractions, thereby reflecting hand gesture activities [1,8]. Force myography (FMG) has been receiving significant interest in complementing sEMG due to its ability to record changes in force or pressure related to muscle volume or muscle stiffness changes without requiring direct skin contact [1]. Jiang et al. [9] utilized an eight-pair armband integrated with eight sEMG sensors and eight force-sensitive resistors (FSR), achieving a 91.6% classification accuracy for American Sign Language digits 0–9. A previous study [10] developed a co-located multi-modal armband incorporating eight pairs of sEMG and pressure-based FMG (pFMG) sensors, which achieved a 94.6% classification accuracy for seven commonly used hand gestures in daily life. Other related research [1,9,10,11,12] demonstrate that co-located multi-modal sensing systems can improve the performance of hand gesture recognition compared to uni-modal sensing systems.

When using wearable sensing systems to assist with prosthetic control, it is essential to minimize the number of sensors while ensuring wearability, comfort and accurate hand gesture classification [5,13]. Furthermore, the limited space within the socket of prosthetic hands restricts the wearable sensing systems with a reduced number of sensors to ensure that they can be easily integrated into the socket of a prosthetic hand [10]. However, current research primarily focuses on increasing the number of sensors to enhance accuracy. Recent research has developed wearable HMI systems that typically employed many sensors [9,10,14,15,16,17]. Despite current research focusing on increasing sensor numbers to enhance accuracy, typically utilizing eight sensors in uni-modal systems to achieve high classification accuracy [18], co-located multi-modal sensing systems offer the potential to reduce space while maintaining a high classification accuracy [11,19]. By strategically optimizing sensor locations and selecting channels with a smaller number from multi-modal sensing systems, it is possible to maintain a high classification accuracy [18].

A degree of noise contamination is frequently inevitable when acquiring non-invasive bio signals [20], potentially affecting their amplitude, temporal and frequency features [21]. Techniques like filtering are commonly employed to mitigate typical noise contaminants but may not consistently achieve complete noise elimination [22]. The quality of bio signals is crucial for accurately extracting gesture-related characteristics, as noise can significantly impact classification outcomes. Hence, bio signals need to contain adequate and sufficient gesture-related information to ensure reliable classification results [20]. Recognizing the critical role of data quality in hand gesture classification, researchers often use Signal-to-Noise Ratio (SNR) as a measurement to evaluate the quality of bio signals [9,10]. For reliable data capture, sEMG sensors must be accurately placed on the muscles, securely attached, and be kept relatively still [21]. However, these conditions change during use [23]. Additionally, the quality of bio signals is closely tied to their acquisition location on the skin surface [24,25]. It is crucial to strategically select sensor location, as incorrect positioning can lead to unreliable results [24]. Therefore, for wearable sensing systems, it is vital to pay attention to the sensor placement to ensure the relevance of the acquired signals from the muscle activities being examined. However, there remains a gap in research regarding the assessment of data quality for channel location selection in hand gesture recognition.

Given that the performance of hand gesture recognition is closely related to data quality, we hypothesize that there is a relationship between hand gesture recognition and data quality across all subjects, provided that the experimental setups and devices are identical. Additionally, due to the space limitations within prosthetic hand sockets, it is necessary to use a limited number of sensors. The results from a previous study [10] demonstrated that using the optimal three-pair of sEMG-pFMG sensors could achieve the best and comparable performance to a complete eight-pair sEMG-pFMG sensing system. Therefore, our goal is to select only three channels/pairs (it is common to use two channels in current prosthetic hands) that can be easily integrated into prosthetic hand systems, enhancing wearability while maintaining reliable performance in hand gesture recognition. To achieve this, we propose two methods to determine the relationship between hand gesture recognition accuracy and data quality to select a reasonable number of channels for an acceptable gesture recognition accuracy: one based on machine learning (ML) techniques and the other based on Pearson’s correlation. Two datasets were used to test these methods: one from an eight-pair co-located sEMG-pFMG armband [10] and another from Ninapro DB2 [26]. By using these methods, we aimed to select three channels/pairs of sensors from the whole eight-channel/pair sensor armband and validate the effectiveness of the methods by comparing their average performance to that of randomly selected three channels/pairs. The aim of this study is not to achieve the state-of-the-art recognition accuracy but to propose a new methodology to select a reasonable number of sensor/data channels to improve the gesture recognition performance of wearable HMI systems consisting of multi-channel sensing systems.

The following work packages are undertaken to deliver the primary contribution of this study:

(1) Comprehensive evaluation of data quality indices: Calculated and analyzed five key data quality indices, including signal-to-noise ratio (SNR), signal-to-motion artifact ratio (SMR), power spectrum deformation ratio (OHM), signal-to-high-frequency noise ratio (SHR), and spectrum maximum-to-minimum drop in power density (DPR) for multiple sensing modalities (sEMG, pFMG, combined sEMG and pFMG).
(2) Comparison of sEMG and pFMG data quality: Compared their data quality indices to demonstrate their complementary nature in handling different types of noise.
(3) Development of ML-based channel selection method: Applied ML techniques to regress the values of each data quality index with the accuracy of corresponding channel combinations, to achieve the automatic selection of the optimal channel combination using the trained ML models.
(4) Development of correlation-based channel selection method: Established the correlation between each data quality index and the accuracy of hand gesture recognition. Developed weighted equations for channel selection by assigning weights to each data quality index based on their correlations with hand gesture recognition accuracy, facilitating the strategic selection of optimal channels for sEMG and pFMG signals.
(5) Validation across different datasets: Validated the effectiveness of the proposed channel selection methods using both the UOW and Ninapro DB2 datasets, demonstrating the generalizability of the methods across different datasets and signal modalities.
(6) Demonstration of practical advantages for wearable HMI systems: Showed that using a reduced number of strategically selected channels (three pairs of sEMG-pFMG sensors) can achieve comparable or even superior performance than a full eight-channel sEMG system, thereby enhancing the practicality and wearability of HMI for prosthetic hand systems.

## 2. Materials and Methods

### 2.1. Datasets

In this study, we used the UOW dataset [10] and the Ninapro DB2 dataset [26]. Given that co-located multi-modal sensing systems are more likely to achieve accurate and reliable hand gesture recognition while reducing the number of sensors to minimize space requirements, our research focuses on evaluating the effectiveness of two proposed channel selection methods using the UOW dataset, which includes combined sEMG-pFMG signals. The Ninapro datasets [26] are one of the largest open-source resources for hand gesture recognition research using sEMG sensors. Various proposed tests with Ninapro datasets [26] have demonstrated its reliability for research purposes. Consequently, the Ninapro DB2 dataset was included in our study to further validate the proposed channel selection methods. All signal processing and model training were performed in Python (version 3.10.11).

**Figure 1:**
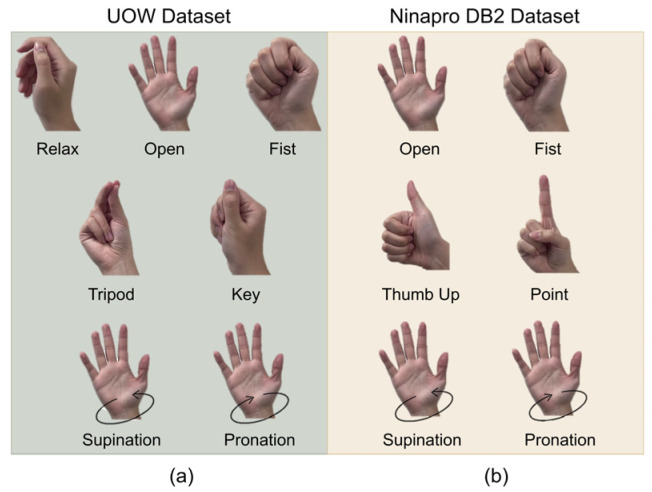
Commonly used hand gestures. (**a**) Gestures in UOW dataset; (**b**) gestures in Ninapro DB2 dataset.

**Figure 2:**
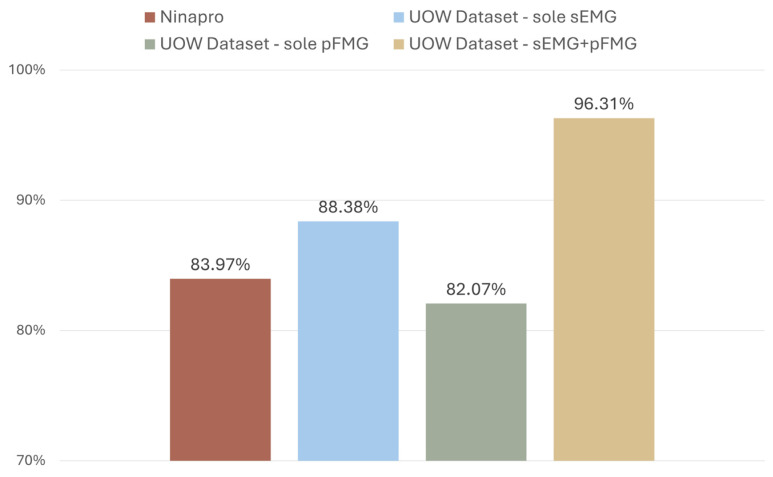
Hand gesture recognition accuracy with different datasets.

The Random Forest (RF) algorithm was chosen to evaluate hand gesture recognition performance on both datasets, due to its proven effectiveness in this area [10]. For the UOW dataset, RF was used to predict seven gestures (Figure 1a) with eight pairs of sEMG-pFMG sensors. For the Ninapro DB2 dataset, which includes a large number of gestures, six commonly used gestures were selected (Figure 1b). These gestures, which focus on daily activities for amputees, were chosen to match the gestures of the UOW dataset, including both wrist and hand gestures. Additionally, data from eight sensors, evenly distributed across the forearm, were utilized to maintain consistency between the two datasets and ensure a more accurate performance evaluation. For the extraction, the integrated absolute value (IAV) was used for pFMG signals. For sEMG signals, the extracted features included the root mean square (RMS), variance (VAR), mean absolute value (MAV), simple integral (SSI), average amplitude change (AAC), and different absolute standard deviation value (DASDV). The choice of these features was followed previously [10], which proved their effectiveness on accurately predicting hand gestures. The sliding window length was set to 200 ms with a 40 ms stride for both the UOW and Ninapro DB2 datasets. This configuration was chosen to maintain consistent preprocessing and ensure the setup is suitable for real-life applications with a response time less than 300 ms [27]. The average accuracies from both datasets using the RF algorithm are shown in Figure 2. The results indicate that for the sole signals, the average accuracies from the UOW dataset with sole sEMG (88.38%), sole pFMG (82.7%), and Ninapro DB2 dataset with sole sEMG (83.97%) are similar. The combined sEMG and pFMG signals achieved the highest accuracy of 96.31%, outperforming the accuracies obtained from using either sEMG or pFMG alone from the UOW dataset.

#### 2.1.1. UOW Dataset

This dataset employed an innovative co-located eight-pair sEMG-pFMG armband to record hand gesture data. They reported that a co-located multi-modal sensing system significantly outperformed sole sEMG and pFMG systems in hand gesture recognition. To further explore how to select three pairs of sEMG-pFMG sensors from the entire 8-pair armband, with the goals of easy integration into the prosthetic socket, enhanced wearability, space efficiency and high performance, we utilized the UOW dataset, which was recorded with the co-located sEMG-pFMG armband. This dataset includes eleven right-hand healthy participants (seven females and four males, 23–35 years old), and the recorded procure was followed the previous study. Ethical approval for this study was granted by the Human Research Ethics Committee at the University of Wollongong.

The UOW dataset includes data from seven commonly used daily gestures: relax, open, fist, tripod, key, supination, and pronation (see Figure 1a). Each gesture was recorded with nine repetitions. To prevent leakage from unused data during training, we separated the repetitions into two datasets: five repetitions as the training dataset and four repetitions as the testing dataset. This approach is more reliable than random shuffling, as data within the same repetition are likely to be highly correlated. The dataset’s size and shape are detailed in Table 1.

#### 2.1.2. Ninapro DB2 Dataset

In this study, we utilized labeled sEMG signals from the Ninapro DB2 dataset [21] to validate the proposed channel selection methods. The data were collected from 40 healthy participants (11 females and 29 males, 23–45 years old). The sEMG data from eight Delsys Trigno (Delsys Inc., Natick, MA, USA), which were evenly attached to each participant’s forearm, were recorded at a sampling rate of 2000 Hz. We selected the data from Exercise B, as it includes more commonly used hand and wrist gestures. From the 17 total gestures in Exercise B, six gestures were chosen for prosthetic control purposes: open, fist, thumb up, point, supination, and pronation (Figure 1b). The relax gesture was omitted from the dataset due to the large number of instances and the inclusion of transient gestures. With six repetitions of each gesture in the dataset, we separated the repetitions into training and testing datasets to prevent data leakage in the training model. Four repetitions were used for training, while the other two repetitions were used for testing.

### 2.2. Data Quality Indices

**Figure 3:**
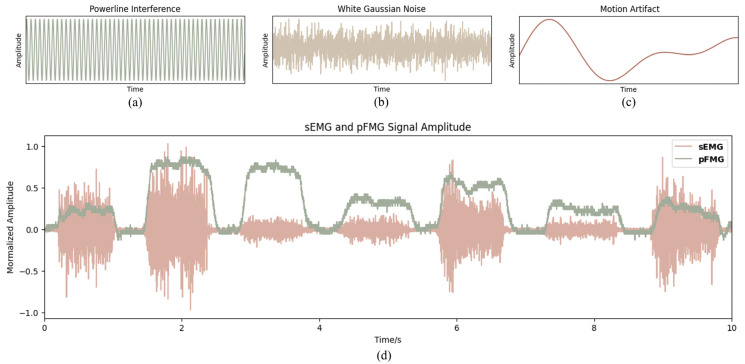
Noise contaminants and bio signals. (**a**) Powerline interference; (**b**) white Gaussian noise; (**c**) motion artifact; (**d**) sEMG and pFMG signals from various hand gestures.

Common noise contaminants include powerline interference (Figure 3a), white Gaussian noise (Figure 3b), and motion artifacts (Figure 3c). Techniques such as filtering are frequently used to reduce these noise contaminants. Figure 3d presents the sEMG and pFMG signals for various gestures. However, some levels of noise remain unavoidable in the recorded signals.

Five data quality indices, SNR, SMR, OHM, SHR and DPR, can be utilized to assess the extent of noise contamination in bio signals from various noise types [16,23]. The SNR typically reflects the level of background noise [8,21]. Therefore, additional indices should be utilized to assess different types of noise, providing a comprehensive evaluation of data quality: SMR quantifiers the proportion of useful signal relative to motion artefacts, helping to evaluate the impact of movement-related noise on data quality; OHM measures the extent to which the power spectrum of signal is distorted by noise, indicating the degree of spectral contamination; SHR quantifies the proportion of signal power relative to upper 20% frequency power, which is considered high-frequency noise; DPR measures the ratio between the maximum and minimum power density values from 13 consecutive points in the frequency spectrum [28], indicating variations in signal strength across all frequencies. The equations for these five data quality indices are [22]

(1) $$SNR=10{\;\log}_{10}{\left(\frac{A_{\mathrm{signal}}}{A_{\mathrm{noise}}}\right)}^{2}=20{\;\log}_{10}\left(\frac{A_{signal}}{A_{noise}}\right)\;$$

(2) $$SMR=10{\;\log}_{10}\left(\frac{P_{signal}}{P_{0–20Hz}}\right)$$

(3) OHM=10 log10(M2M02M1M0)

(4) $$SHR=10{\;\log}_{10}\left(\frac{P_{signal}}{P_{upper20\%}}\right)$$

(5) $$DPR=10{\;\log}_{10}\left(\frac{{MPD}_{highest}}{{MPD}_{lowest}}\right)$$

where *A_noise_* is the root mean square (RMS) of the resting signal, *A_signal_* is the RMS of the active muscle signal, $P_{signal}$ represents the total signal power, $P_{0\text{–}20Hz}$ denotes the total signal power within the 0–20 Hz range, $P_{upper20\%}$ is the total signal power in the upper 20% frequency range, $M_{n}$ indicates the $n^{th}$ order spectral moments, and *MPD* refers to the mean power density of 13 consecutive points shifted across all frequencies.

The five data quality indices for the UOW dataset are presented in Table 2, with the values representing the average across all subjects. From the results, the higher SNR for pFMG suggests that pFMG is less sensitive to ambient noise and provides a cleaner signal compared to sEMG. Conversely, the higher SMR indicated that sEMG should be more resistant to motion artefacts. Additionally, a higher SHR for sEMG implies that it is less affected by high-frequency noise. In contrast, FMG sensors, with their lower bandwidth (0–10 Hz) [29], are more impacted by high-frequency noise, making the SHR lower for pFMG. A higher DPR for pFMG indicates that the pFMG signal has a more distinct and pronounced frequency component compared to noise contaminants. Overall, both sEMG and pFMG signals offer unique advantages in handling different types of noise. Therefore, combining these two modalities can enhance the overall signal quality.

### 2.3. Channel Selection

**Figure 4:**
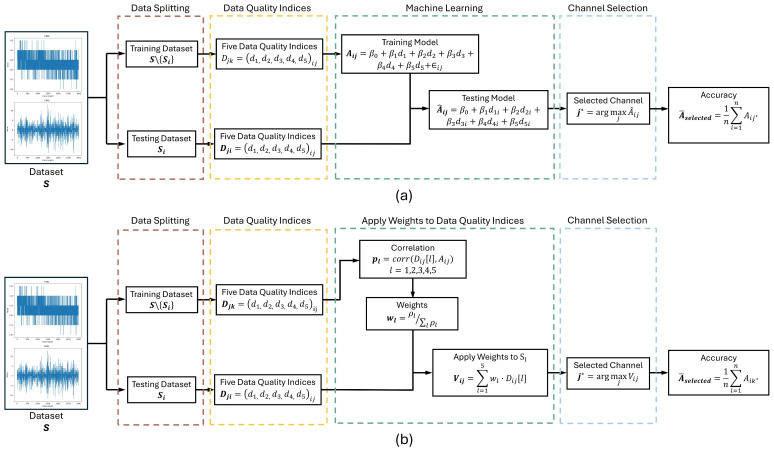
Channel selection methods. (**a**) ML-based method; (**b**) correlation-based method.

Given the critical importance of data quality in bio signals for hand gesture recognition performance, we propose two methods for channel selection using data quality indices. By recording signals from an armband with eight evenly distributed channels/pairs on the arm, we calculated data quality values from five data quality indices for each channel. Our goal was to select three channels/pairs from the entire armband to save space and achieve easier integration with prosthetic systems. To achieve this, we listed all possible combinations of three channels/pairs and their corresponding values for each signal quality index. Both methods employed the leave-one-subject-out approach, based on the hypothesis that a general relationship exists between data quality and hand gesture recognition accuracy across all subjects under consistent experimental conditions. The first method (Figure 4a) utilized ML techniques to identify this relationship, using the five data quality indices as features and the gesture recognition accuracy of each three-channel/pair combination from the RF algorithm as the target values. By creating the regression model, we could predict the channel/pair combination that would likely yield the highest hand gesture recognition accuracy for the remaining subject when new feature data were input into the trained model. The second method (Figure 4b) involved calculating the correlation between each data quality index and the actual accuracies obtained from the RF algorithm. These correlations were then transformed into weights. These weights were applied to the data index values for the remaining subject. After applying the weights, the values were summed, and the channel/pair combination with the highest sum was chosen. This combination of three channels/pairs was considered to have the potential to provide the best hand gesture recognition accuracy. The computational complexity of the proposed channel selection strategy is low. Both the ML-based and correlation-based methods involve simple statistical operations and linear modeling, and the channel selection procedure can be performed offline prior to online gesture recognition.

By comparing the average accuracy between selected channels/pairs and randomly chosen channels among all subjects, it can demonstrate the potential improvement in accuracy achieved through the strategic channel selection. Both the UOW and Ninapro DB2 datasets were tested with these methods. For the UOW dataset, our goal was to identify the optimal three pairs of sEMG-pFMG sensors that deliver high performance. By strategically selecting channels from a small number of co-located multi-modal sensors, it can enhance wearability and reduce the complexity of the sensing system while maintaining high performance. For the Ninapro DB2 dataset, which includes only sEMG signals, we utilized it to validate the effectiveness of the two proposed channel selection methods. This validation helps ensure that the proposed methods are applicable not only to the UOW dataset but also to the datasets with the same hand gesture recognition purpose.

#### 2.3.1. ML-Based Channel Selection

Both linear and non-linear learning models, including Linear Regression (LR), Support Vector Machine (SVM) and Random Forest (RF), were examined during an initial analysis to inform the design of the ML-based channel selection strategy. This preliminary analysis indicated that linear modeling was sufficient to capture the dominant relationship between data quality indices and recognition performance. Please note that the use of LR does not imply an assumption that the relationship between data quality indices and recognition accuracy is strictly linear. Instead, LR is employed as a practical modeling tool. The ML-based channel selection employed an LR model to predict the accuracy of all combinations by selecting three from eight channels/pairs based on five data quality indices. This method adopted a leave-one-subject-out cross-validation approach to ensure robustness and generalizability. For each subject $S_{i}$ in the set of subjects $S=\left\{S_{1},S_{2},\ldots ,S_{n}\right\}$, the remaining subjects $S\backslash \left\{S_{i}\right\}$ were used to train the LR model.

Firstly, let $D_{ij}={\left(d_{1},d_{2},d_{3},d_{4},d_{5}\right)}_{ij}$ represent the vector of five data quality indices for the $j^{th}$ channel combination (selecting three from eight) of the $i^{th}$ subject, and let $A_{ij}$ represent the corresponding accuracy. The LR model aimed to establish a relationship between the data quality indices and the accuracy using the following equation:

(6) $$A_{ij}=\beta_{o}+\beta_{1}d_{1}+\beta_{2}d_{2}+\beta_{3}d_{3}+\beta_{4}d_{4}+\beta_{5}d_{5}+\in_{ij}$$

where $\beta_{o}$ is the intercept, $\beta_{1},\beta_{2},\beta_{3},\beta_{4},\beta_{5}$ are the regression coefficients associated with each data quality index, and $\in_{ij}$ is the error term. The coefficients are estimated using the training set $S\backslash \left\{S_{i}\right\}$.

Once the model was trained, it was used to predict the accuracy for each channel combination of the left-out subject $S_{i}$. The predicted accuracy for each combination was given by

(7) $${\hat{A}}_{ij}=\beta_{o}+\beta_{1}d_{1i}+\beta_{2}d_{2i}+\beta_{3}d_{3i}+\beta_{4}d_{4i}+\beta_{5}d_{5i}$$

where $d_{1i},d_{2i},d_{3i},d_{4i},d_{5i}$ are the data quality indices for the left-out subject $S_{i}$.

Among all channel/pair combinations, the one with the highest predicted accuracy was selected as the best channel/pair combination, defined as

(8) $$j^{*}=\arg\underset{j}{\max}{\hat{A}}_{ij}$$

The real accuracy $A_{ij^{*}}$ of the selected channel/pair combination $j^{*}$ was calculated using the RF algorithm. This process was repeated for each subject, ensuring that the data from every subject were used as the test set once. To evaluate the effectiveness of this ML-based channel selection method, the average accuracy of the selected channel/pair across all subjects was calculated from

(9) $${\bar{A}}_{selected}=\frac{1}{n}\sum_{i=1}^{n}A_{ij^{*}}$$

For comparison, the average accuracy ${\bar{A}}_{random}$ was also calculated with the RF algorithm for randomly selected combinations of three channels/pairs for each subject. The average accuracy from random selection was used as a baseline to assess the hand gesture recognition accuracy from the selected channel/pair achieved by the proposed method.

#### 2.3.2. Correlation-Based Channel Selection

The correlation-based channel selection utilized the statistical Pearson’s correlation [30] between data quality indices and accuracy to identify the optimal channel combinations. Pearson’s correlation coefficient is defined as follows [30]:

(10) $$corr=\;\frac{\sum_{i=1}^{n}\left(x_{i}-\bar{x}\right)\left(y_{i}-\bar{y}\right)}{\sqrt{\sum_{i=1}^{n}{\left(x_{i}-\bar{x}\right)}^{2}\sum_{i=1}^{n}{\left(y_{i}-\bar{y}\right)}^{2}}}$$

where *corr* is the Pearson’s correlation coefficient, n is the number of samples in the dataset, and *x* and *y* are the two variables.

This method also employed a leave-one-subject-out cross-validation technique to ensure its effectiveness. For each subject $S_{i}$, the method evaluated all possible combinations of three channels/pairs from a total of eight channels/pairs. Let $D_{ij}={\left(d_{1},d_{2},d_{3},d_{4},d_{5}\right)}_{ij}$ represent the values of the five data quality indices for the $j^{th}$ channel combination (selecting three from eight) of the $i^{th}$ subject. The corresponding accuracy for each combination was represented as $A_{ij}$. The method is to use the data from the remaining subjects $S\backslash \left\{S_{i}\right\}$ to compute the correlations between each data quality index and the associated accuracy. The correlation coefficient $\rho_{l}$ for each data quality index $D_{l}$ with the accuracy *A* was computed as follows:

(11) $$\rho_{l}=corr\left(D_{ij}\left[l\right],\;A_{ij}\right),\;\;\;\;l=1,2,3,4,5$$

This coefficient measures the strength and direction of the linear relationship between each data quality index and the accuracy. This significance of each correlation is tested using *p*-values [31]. Statistically significant correlations are transformed into weights $w_{l}$ reflecting their importance in predicting accuracy, while non-significant correlations are assigned a weight of zero. This ensures that only the significant and statistically reliable correlations are considered in the channel selection process, enhancing the reliability of the selected channel combinations. The weights $w_{l}$ were calculated as follows:

(12) wl={ρl∑lρl,  if the p-value of ρl <0.050,  otherwise

For the left-out subject $S_{i}$, these weights $w_{l}$ were then applied to the data quality indices $D_{ij}$ to compute a weighted score $V_{ij}$ for each channel combination:

(13) $$V_{ij}=\sum_{l=1}^{5}w_{l}\cdot D_{ij}\left[l\right]$$

The channel combination $j^{*}$ that achieved the highest weighted score $V_{ij}$ was selected as the best channel/pair combination, defined as

(14) $$j^{*}=\arg\underset{j}{\max}V_{ij}$$

The real accuracy $A_{ij^{*}}$ of the selected channel/pair combination $j^{*}$ was calculated using the RF algorithm. This process was repeated for each subject, ensuring that the data from every subject were used as the test set once. To evaluate the performance of this correlation-based channel selection method, the average accuracy ${\bar{A}}_{selected}$ was calculated (see Equation (9)). A baseline ${\bar{A}}_{random}$ was determined by calculating the average accuracy for randomly selected combinations of three channels/pair. The comparison between ${\bar{A}}_{selected}$ and ${\bar{A}}_{random}$ was used to assess whether this method provided an improvement for hand gesture recognition accuracy.

## 3. Results and Discussion

The evaluation of channel selection methods across the UOW dataset and Ninapro DB2 dataset demonstrates the effectiveness of different methods in optimizing hand gesture recognition performance when using a limited number of channels/pairs. The five selected data quality indicators were chosen to capture complementary aspects of signal integrity across both sEMG and pFMG modalities. The SNR primarily reflects background noise levels and overall signal strength, which is critical for electrical sEMG recordings. The SMR explicitly quantifies motion-induced artifacts, which are particularly relevant for wearable sensing under dynamic conditions and have distinct manifestations in mechanical pFMG signals. In the frequency domain, the OHM and SHR characterize spectral distortion and high-frequency noise contamination, respectively, which can arise from electrode–skin impedance variations in sEMG and mechanical vibration or sensor deformation in pFMG. DPR further captures dynamic variations in spectral power distribution, reflecting signal instability caused by movement or load changes. Importantly, due to the fundamentally different electrical and mechanical characteristics of sEMG and pFMG signals, reliance on a single data quality metric is insufficient. The joint use of multiple complementary DQIs enables a more comprehensive assessment of signal quality and facilitates effective channel selection in multi-modal fusion scenarios, thereby improving gesture recognition performance. The results demonstrate notable differences in performance between the two channel selection methods and random channel selection.

**Figure 5:**
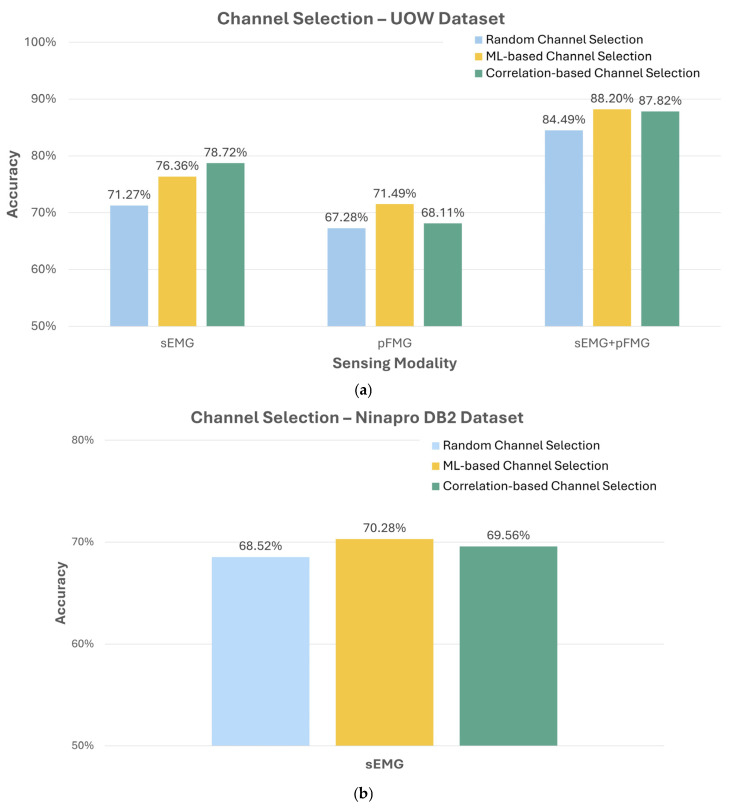
Hand gesture recognition accuracy for two datasets with different channel selection methods. (**a**) Results from the UOW dataset, including sole sEMG signals, sole pFMG signals, and fused sEMG + pFMG signals. (**b**) Results from the Ninapro DB2 dataset using sEMG signals only. For each dataset, performance is reported for random channel selection, correlation-based channel selection, and the ML-based channel selection method.

For the UOW dataset, which includes both sEMG and pFMG signal, the performance of the channel selection methods is shown in Figure 5a. The ML-based channel selection method achieved 76.36% accuracy with sole sEMG signals, 71.59% accuracy with sole pFMG signals, and 88.2% accuracy with combined sEMG-pFMG signals. The correlation-based selection method achieved 78.72% accuracy with sole sEMG signals from, 68.11% accuracy with sole pFMG signals, and 87.82% accuracy with combined sEMG-pFMG signals. Both channel selection methods consistently exceeded the average hand gesture recognition accuracy achieved by randomly selecting channel/pairs, for both sEMG and pFMG signals, as well as the combined signals. The ML-based channel selection method achieved the highest accuracy with sole sEMG signals and combined sEMG-pFMG signals, while the correlation-based channel selection method delivered the highest accuracy for the pFMG signal. Overall, the ML-based channel selection method proved to be slightly more effective than the correlation-based method. However, both methods enhanced the hand gesture recognition performance compared to random selection, indicating their advantage in identifying valuable channel combinations. The Ninapro DB2 dataset, which includes only sEMG signals, was used to validate the effectiveness of the two channel selection methods. The performance of these channel selection methods for the Ninapro DB2 dataset is shown in Figure 5b. Both ML-based and correlation-based channel selection methods showed improvements over random channel selection, with ML-based method (70.28% accuracy) yielding better results. The results from the Ninapro DB2 dataset align with those from the UOW dataset, indicating that both channel selection methods enhance hand gesture recognition performance.

To assess whether the performance improvements achieved by the proposed channel selection methods over random selection are statistically significant, paired *t*-tests were conducted across subjects. The results demonstrate statistically significant improvements for all evaluated cases, including combined sEMG-pFMG signals (*p* = 0.037), sole sEMG signals (*p* = 0.030), sole pFMG signals (*p* = 0.047), and the Ninapro DB2 dataset (*p* = 0.007). These findings confirm that the observed accuracy gains are unlikely to arise from random variation. The more pronounced statistical significance observed for the Ninapro DB2 dataset is attributed to its larger sample size, which increases the statistical power.

Additionally, when using the three pairs of sEMG-pFMG sensors selected through the ML-based channel selection method, the performance is comparable to that of using the entire eight-channel sole sEMG systems. Specifically, the average accuracy with the selected sEMG-pFMG sensing pairs achieved an impressive accuracy of 88.2% (Figure 5a). This closely aligned with the 88.38% accuracy (Figure 2) achieved with the UOW dataset when utilizing the entire eight-channel sole sEMG signals. These results highlight the strength of the ML-based channel selection method in optimizing sensor placement. By strategically selecting channels, it is possible to reduce the number of sensors while maintaining high accuracy, thereby enhancing the practicality and wearability of the HMI sensing system. This reduction in sensors can lead to simpler, more efficient, and potentially easier integrated prosthetic sensing systems with promising performance.

**Figure 6:**
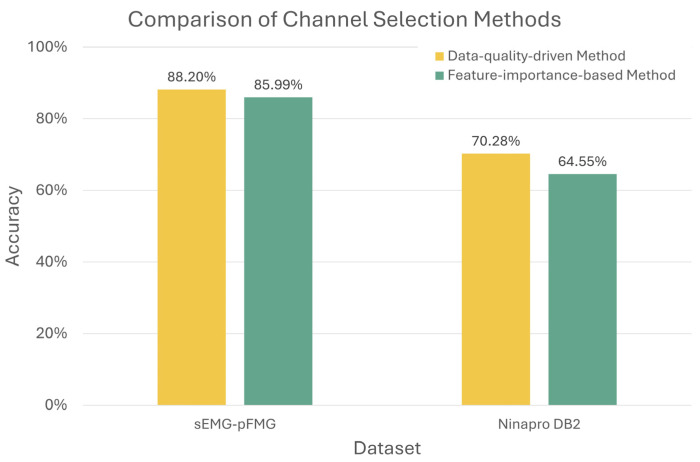
Comparison of channel selection methods for hand gesture recognition accuracy across datasets. Results are reported for two datasets: the UOW sEMG-pFMG dataset and the public Ninapro DB2 dataset. The comparison includes the proposed data-quality-driven channel selection method and a feature-importance-based method.

To further evaluate the effectiveness and generalizability of the proposed data-quality-driven channel selection method, a comparison with a widely used feature-importance-based channel selection baseline was conducted on both the fused sEMG-pFMG dataset and the public Ninapro DB2 dataset (Figure 6). For the fused sEMG-pFMG dataset, the feature-importance-based method achieved an average recognition accuracy of 85.99%, whereas the proposed data-quality-driven method achieved a higher accuracy of 88.20%. Similarly, on the Ninapro DB2 dataset, the feature-importance-based method yielded an average accuracy of 64.55%, compared to 70.28% achieved by the proposed method. Notably, on the DB2 dataset with a larger number of subjects, the feature-importance-based method even underperformed random channel selection (67.28%), highlighting its sensitivity to inter-subject variability. It is important to note that feature-importance-based channel selection methods in EMG-based gesture recognition are typically computed using data from the same subject [32,33]. In contrast, the proposed data-quality-driven method leverages signal quality characteristics that are closely linked to the physical properties of the recorded signals, enabling more robust channel selection across subjects. This highlights its suitability for practical prosthetic control and wearable HMI applications, where calibration data per subject are limited, yet robustness and generalizability across users are critical.

To examine whether the proposed channel selection strategy depends on the underlying modeling tool, a preliminary comparison between different relationship modeling approaches was conducted. Specifically, linear (LR) and non-linear (RF and SVM) were employed to establish the relationship between data quality indices and recognition accuracy using the same sEMG-pFMG dataset. For the linear approach, the LR method achieved average recognition accuracies of 88.20% for fused sEMG-pFMG signals, 76.36% for sole sEMG signals, and 71.49% for sole pFMG signals (Figure 5a). For the non-linear approach, RF achieved an average recognition accuracy of 87.18% for fused sEMG-pFMG signals, and 85.16% for SVM. For single-modality signals, RF achieved accuracies of 77.56% and 70.82% for sEMG and pFMG, respectively, while SVM yielded 74.47% for sEMG and 66.38% for pFMG. Additionally, the reported correlation-based approach (87.82% for fused sEMG-pFMG, 78.72% for sEMG signals, and 68.11% for pFMG signals.) does not rely on any classifier or learning model. These results indicate that the resulting channel selection trends remain consistent across these different approaches. This demonstrates that the effectiveness of the proposed data-quality-driven channel selection strategy is not an artifact of a specific classifier or modeling tool but instead reflects the robustness of the underlying relationship between data quality and recognition performance. From a signal processing perspective, data quality indices are designed to quantify the signal integrity, including the noise level, stability, and artifact contamination. Improvements in these measures generally correspond to improved recording conditions and recognition performance in a monotonic manner. While the underlying relationship may exhibit non-linear characteristics in certain cases, the results suggest that linear modeling provides a sufficient and computationally efficient approximation for channel selection in wearable HMI systems. Additionally, from a practical perspective, the proposed channel selection framework does not impose an additional computational burden during online gesture recognition. Once the optimal channel subset is determined, the subsequent real-time inference operates on a reduced number of channels using standard classifiers. Therefore, the proposed method is compatible with real-time wearable HMI systems and prosthetic control applications, where low latency and computational efficiency are critical.

## 4. Conclusions and Future Work

This study presents a thorough evaluation of two channel selection methods to enhance the hand gesture recognition performance by using reduced number of sensors (three pairs/channels) with both sEMG and pFMG signals. The findings underscore the effective contribution of ML-based and correlation-based channel selection methods in enhancing hand gesture recognition accuracy compared to random channel selection. In particular, the ML-based channel selection method demonstrated superior performance, particularly with combined sEMG-pFMG signals, achieving comparable results to a full eight-channel sEMG system while using fewer sensors. This highlights the potential for more practical and wearable HMI systems through strategic channel selection by reducing the number of sensors and optimizing sensor placement. The ability to maintain a high hand gesture recognition accuracy with a reduced number of sensors offers substantial benefits in terms of system simplicity, efficiency, wearability, and cost-effectiveness, making it highly suitable for real-world HMI prosthetic application.

The results of this study offer valuable methods for strategically selecting channels, demonstrating that it is possible to achieve a high hand gesture recognition performance with fewer sensors. Despite these advancements, there are remaining limitations and opportunities for further improvement:

(1) Validation with larger and more diverse datasets: The current study is limited by a relatively small sample size, which is due to challenges in obtaining larger datasets from human participants. Future research should aim to address this limitation by incorporating larger and more varied datasets.
(2) Real-time implementation: This study evaluates the proposed channel selection methods using offline datasets, where channel selection is performed prior to the HGR task. Future work will focus on developing and testing real-time implementations that deploy the selected channel subsets in online HMI and prosthetic control scenarios, in order to further assess the robustness, adaptability, and practical effectiveness.
(3) Integration with other sensing modalities: Investigating the integration of sEMG and pFMG with other sensing modalities may provide more comprehensive data and improve hand gesture recognition performance.
(4) Exploration of additional channel selection methods: This study primarily focuses on utilizing data quality indices to assist in channel selection. Further research could investigate alternative methods to complement the proposed methods, aiming to further enhance the channel selection process and potentially improve hand gesture recognition accuracy.
(5) Sensor displacement and inter-session variability: Future work will explicitly evaluate the proposed data-quality-driven channel selection framework under sensor displacement and inter-session scenarios to further assess its robustness in real-world use cases.
(6) Adaptive data quality indicators: Future work will investigate alternative or adaptive data quality indicators to further assess the robustness of the proposed channel selection framework under varying signal conditions.

This study enhances our understanding of how data quality indices can be effectively used for channel selection and their impact on hand gesture recognition accuracy. This research represents a pioneering effort to apply both ML-based and correlation-based methods for optimizing sensor placement in wearable HMI systems. By demonstrating the potential of these methods, the study offers valuable insights and suggests new ideas for researchers to explore more reliable and robust channel selection methods.

## Figures and Tables

**Table 1:** Summary of dataset structure.

Dataset	Gestures	Repetitions	Channel	Data Shape
UOW dataset	7	9	8 + 8	(18,000, 7, 16)
Training	7	5	8 + 8	(10,000, 7, 16)
Testing	7	4	8 + 8	(8000, 7, 16)

**Table 2:** Values of data quality indices in UOW dataset.

Data Quality Index	sEMG	pFMG
SNR	13.73	18.74
SMR	34.88	15.43
OHM	0.53	0.72
SHR	23.62	13.84
DPR	36.5	44.57

## Data Availability

The datasets generated and/or analyzed during the current study are not publicly available due to ethical considerations and participant privacy, and because they form part of ongoing research. The data can be requested from the corresponding author for non-commercial academic purposes.

## Abbreviations

The following abbreviations are used in this manuscript:

AAC	Average amplitude change
DASDV	Different absolute standard deviation value
DPR	Spectrum maximum-to-minimum drop in power density
FMG	Force myography
FSR	Force-sensitive resistors
HMI	Human–machine interface
IAV	Integrated absolute value
MAV	Mean absolute value
ML	Machine learning
MUAP	Motor unit action potential
OHM	Power spectrum deformation ratio
pFMG	Pressure-based force myography
RF	Random forest
RMS	Root mean square
sEMG	Surface electromyography
SHR	Signal-to-high-frequency noise ratio
SMR	Signal-to-motion artifact ratio
SNR	Signal-to-noise ratio
SSI	Sample integral
SVM	Support vector machine
VAR	Variance
